# Ultraviolet-B Radiation Represses Primary Root Elongation by Inhibiting Cell Proliferation in the Meristematic Zone of Arabidopsis Seedlings

**DOI:** 10.3389/fpls.2022.829336

**Published:** 2022-03-24

**Authors:** Maria Luján Sheridan, Lucio Simonelli, Marisol Giustozzi, Paula Casati

**Affiliations:** Centro de Estudios Fotosintéticos y Bioquímicos, Universidad Nacional de Rosario, Rosario, Argentina

**Keywords:** cell elongation, cell proliferation, primary root, programmed cell death, UV-B radiation

## Abstract

In *Arabidopsis thaliana* plants, exposure to UV-B induces an inhibition of primary root elongation. Different mutants have been isolated that are deficient in this response; however, little is known about the cellular and molecular mechanisms that regulate inhibition of root elongation in seedlings exposed to UV-B. In this work, we investigated the effect UV-B irradiation of different organs on primary root elongation. Our results demonstrate that irradiation of the leaves and shoots only induce a partial inhibition of primary root elongation, while when only roots are exposed to this radiation, primary root inhibition is similar as that measured when the complete seedling is irradiated. The consequences of exposure at different root developmental stages and times after the end of the treatment was also studied. We here show that inhibition of primary root elongation is a consequence of a decrease in cell proliferation in the meristematic zone of the primary roots, while the elongation zone size is not affected by the treatment. The decrease in cell number after UV-B exposure is partially compensated by an increase in cell length in the root meristem; however, this compensation is not enough to maintain the meristem size. We also here demonstrate that, similarly as what occurs in developing leaves, GROWTH REGULATING FACTOR 3 (GRF3) transcription factor regulates cell proliferation in UV-B irradiated roots; however, and in contrast to what occurs in the leaves, this response does not depend on the presence of MITOGEN ACTIVATED PROTEIN KINASE 3 (MPK3). Inhibition of primary root elongation by UV-B under our experimental conditions is also independent of the UV-B photoreceptor UV RESISTANT LOCUS 8 (UVR8) or ATAXIA TELANGIECTASIA MUTATED (ATM); but a deficiency in *ATM AND RAD3-RELATED* (*ATR*) expression increases UV-B sensitivity in the roots. Finally, our data demonstrate that UV-B affects primary root growth in various Arabidopsis accessions, showing different sensitivities to this radiation.

## Introduction

Plants are continually exposed to a changing environment, and this influences their developmental programs. Light is probably the most important environmental factor that affects plant growth and development. Ultraviolet-B (UV-B) radiation (280–315 nm) represents only a small proportion of the solar radiation that reaches the Earth; however, plants respond to exposure to wavelengths in the UV-B range, for example showing changes in plant morphology, physiology and producing secondary metabolites (Dotto and Casati, 2017).

Ultraviolet-B radiation effects on plant growth include specific photomorphogenic responses, mostly mediated by the UV-B specific photoreceptor UV RESISTANT LOCUS 8 (UVR8), and non-specific stress or genotoxic responses (Hu et al., 2016; Podolec et al., 2021). UVR8 is so far the only UV-B specific photoreceptor identified in plants (for a review, see Podolec et al., 2021), this receptor modulates plant responses to UV-B interacting with CONSTITUTIVELY PHOTOMORPHOGENIC 1 (COP1) after UV-B exposure to activate transcription of UV-B responsive genes, initiating UV-B acclimation pathways. On the other hand, the non-specific effects include the formation of reactive oxygen species (ROS), DNA damage and the activation of the DNA damage response, membrane changes and protein crosslinking, these effects are usually produced after exposure to high UV-B doses or long exposure times (Manova and Gruszka, 2015; Parihar et al., 2015). These high UV-B activated responses are usually independent of UVR8 and involve signaling pathways that are partially conserved among different organisms (Hu et al., 2016). One of these pathways is the DNA damage response (DDR), which is induced after DNA damage; in this pathway the protein kinases ATAXIA TELANGIECTASIA MUTATED (ATM) and ATM AND RAD3-RELATED (ATR) function as a key regulators of the DNA damage response, activating SUPPRESSOR OF GAMMA RESPONSE 1 (SOG1; Furukawa et al., 2010), a transcription factor that regulates the expression of genes that encode proteins in this pathway (Bourbousse et al., 2018). A second UV-B pathway that acts independently of UVR8 requires the action of mitogen activated protein kinases (MAPK) MPK3 and MPK6, which are also triggered after exposure to DNA-damaging agents in Arabidopsis; thus, the MPK3/MPK6 cascade is an additional important pathway contributing to UV-B-induced DNA damage (González Besteiro and Ulm, 2013; Hu et al., 2016).

One plant response to UV-B is the reduction in the leaf area (Dotto and Casati, 2017). This decrease can be a consequence of an inhibition of cell division and/or to a decrease in cell expansion, and these different effects are due to different experimental conditions, developmental stages or plant species. For example, UV-B conditions that produce DNA damage in general inhibit cell proliferation, while lower doses and/or chronic UV-B exposure can produce both inhibition of cell proliferation and expansion. In Arabidopsis, the decrease in leaf size in UV-B irradiated plants is a consequence of inhibition of cell proliferation mediated by the microRNA miR396, which downregulates the expression of *GROWTH REGULATING FACTORS* (*GRF*) transcription factors (Casadevall et al., 2013). miRNA396 is upregulated by UV-B in proliferating leaves, and this induces the decrease in *GRF1*, *GRF2*, and *GRF3* levels. Induction of miR396 results in an inhibition of cell proliferation without affecting cell expansion. Interestingly, this response is independent of the UVR8 photereceptor and ATR, but depends on the presence of MPK3 (Casadevall et al., 2013). Interestingly, the role of miR396 in primary root elongation after UV-B exposure was also demonstrated (Gómez et al., 2019). Arabidopsis transgenic plants expressing an artificial target mimic directed against miR396 (*MIM396*) and showing a decrease in the endogenous microRNA activity, had a shorter primary root than WT plants, but they showed a lower inhibition of primary root elongation after UV-B exposure. In this way, inhibition of primary root elongation in Arabidopsis seedlings by UV-B is also regulated by this microRNA.

In addition, different reports have shown that Arabidopsis plants have a UV-B-sensing mechanism in the roots that regulate morphogenesis (Tong et al., 2008; Leasure et al., 2009). Moreover, after UV-B exposure at high intensity, in Arabidopsis roots, there is an upregulation of the expression of cell cycle regulatory and DNA damage genes, suggesting that UV-B-induced DNA damage in this organ may induce a delay of the G1-to-S transition of the cell cycle (Jiang et al., 2011). Thus, this delay in the progression of the cell cycle may be a protective mechanism to prevent cells with damaged DNA from dividing. Despite this, little is known about the cellular and molecular mechanisms that regulate the inhibition of root elongation in seedlings exposed to UV-B. Here, we investigated the effect UV-B irradiation of different organs on primary root elongation. We also analyzed the consequences of exposure at different root developmental stages and times after the end of the treatment. Our data show that inhibition of primary root elongation is a consequence of an inhibition of cell proliferation in the meristematic zone of the primary roots, while the elongation zone size is not affected by the treatment. The decrease in cell number after UV-B exposure is partially compensated by an increase in cell length in the root meristem; however, this compensation is not enough to maintain the meristem size of control roots grown in the absence of UV-B. The role of GRF3 was also analyzed, demonstrating that similarly as what occurs in developing leaves, transcription factors from this family regulate cell proliferation in the roots under UV-B conditions; however, and in contrast to what occurs in the leaves, this response does not depend on the presence of MPK3. We here also demonstrate that inhibition of primary root elongation by UV-B under the conditions of our experiments is independent of the UV-B photoreceptor UVR8 or ATM; but a deficiency in *ATR* expression significantly increases UV-B sensitivity in the roots. Finally, our data demonstrate that UV-B affects primary root growth in various Arabidopsis accessions, showing different sensitivities to this radiation.

## Materials and Methods

### Plant Material, Growth Conditions, and Irradiation Protocols

*Arabidopsis thaliana* ecotypes Columbia (accessions Col-0, Col-3, and Col-4), Landsberg erecta (Ler) and Wassilewskija (Ws) were used for all experiments. *uvr8*, *mpk3*, *atm*, and *atr* seeds were a gift from Roman Ulm (University of Geneva, Switzerland). *rGRF3* plants were previously described by Rodriguez et al. (2010). *msh6* mutants in a Col-0 background were previously described in Lario et al. (2011).

For all the experiments, Arabidopsis seeds were grown on Murashige and Skoog (MS) growth medium supplemented with 0.7% agar in Petri dishes, and they were kept in a vertical position in the growth chamber at 22°C under a 16 h/8 h light/dark photoperiod (100 μEm^–2^ s^–1^). In most experiments, 5 days after germination, full seedlings were irradiated for 1 h using UV-B lamps on fixtures mounted 30 cm above the plants (9 μmol m^–2^ s^–1^ UV-B and 2.9 μmol m^–2^ s^–1^ UV-A, Bio-Rad ChemiDoc™ XRS UV-B lamps, catalog 1708097). These lamps have a peak at 302 nm and an emission spectra from 290 to 310 nm, and they were covered using cellulose acetate filters (CA, 100 mm extra-clear cellulose acetate plastic, Tap Plastics, Mountain View, CA, United States). The CA filter absorbs wavelengths lower than 290 nm; this control was done in case some lower wavelength radiation was produced with lamps aging. As a control without UV-B, plants were exposed for the same time under the lamps also covered with a polyester plastic that absorbs UV-B at wavelengths lower than 320 nm (PE, 100 mm clear polyester plastic; Tap Plastics). UV radiation was recorded using a UV-B/UV-A radiometer (UV203 AB radiometer; Macam Photometrics). Alternatively, during the UV-B treatment, the roots or the aerial parts of the seedlings (cotyledons and hypocotyl) were covered using a black paper, so as that only some parts of the seedlings are UV-B irradiated. After the UV-B treatment, seedlings were kept in the growth chamber under a 16 h/8 h light/dark photoperiod in the absence of UV-B. Alternatively, control and UV-B treated seedlings were kept in the dark to analyze the effect of photoreactivation in primary root elongation.

For fluence response analysis of primary root elongation, seedlings were irradiated for 1 h as described above at 4.5, 6.75, 9, or 11.25 μmol m^–2^ s^–1^ UV-B. After the UV-B treatment, seedlings were kept in the growth chamber under a 16 h/8 h light/dark photoperiod in the absence of UV-B.

### Primary Root Length Measurements

Seedlings were grown for 5 or 9 days in MS-agar plates, they were UV-B irradiated as described above and they were then kept in the growth chamber in the absence of UV-B for 4 days. Plates were photographed before the treatment and 1, 2, 3, and 4 days after. Primary root length was measured using the ImageJ software version 1.52p.

### Primary Root Meristem Analysis and Programmed Cell Death After Ultraviolet-B Exposure

Five days after stratification, MS-agar plate grown seedlings were irradiated with UV-B or kept under control conditions. Then, seedlings were maintained in the growth chamber under control conditions without UV-B for 24 or 96 h, and meristem length, cell number, average cell length in the meristematic zone and programmed cell death (PCD) were quantified by staining the root tips with a modified pseudo-Schiff propidium iodide (PI) staining protocol (Furukawa et al., 2010). Primary roots were analyzed by confocal laser scanning microscopy (Nikon C1) under water with 40X. The excitation wavelength for PI-stained samples was 488 nm and emission was collected at 520–720 nm. The meristematic zone characteristics were analyzed using the Image J software version 1.52p.

### DNA Damage Analysis

Cyclobutane pyrimidine dimers (CPD) were measured using monoclonal antibodies by dot-blot analysis (TDM-2; Cosmo Bio Co., Ltd., Japan). Twelve-day-old plants were treated with UV-B for 1 or 4 h as described above, and samples (0.1 g) were collected after the treatment. 2 μg of the extracted DNA by a modified cetyltrimethylammonium bromide (CTAB) method was then denatured using 0.3 M NaOH for 10 min. Samples were analyzed using a nylon membrane (PerkinElmer Life Sciences, Inc.) in sextuplicate. The membrane was incubated at 80°C for 2 h and blocked with a buffer containing 20 mM Tris–HCl, pH 7.6, 137 mM NaCl (TBS) and 5% (p/v) dried milk for 1 h at room temperature. The membrane was finally washed with TBS and incubated with anti-CPDs antibodies (1:2000 in TBS) overnight at 4°C with agitation. Unbound antibodies were washed away and secondary antibodies conjugated to alkaline phosphatase (1:3000; Bio-Rad) were added. The blot was washed and finally developed by the addition of 5-bromo-4-chloro-3-indolyl phosphate and nitroblue tetrazolium. Dots were quantified by densitometry using ImageQuant software version 5.2. Total DNA was quantified fluorometrically using the Qubit dsDNA assay kit (Invitrogen).

### Statistical Analysis

Comparisons between one independent variable were done using One-Way ANOVA (Dunn Test), while comparisons between more than two variables were analyzed using Two-Way ANOVA (Tukey’s Test), using non-transformed data. These statistical analyses were performed using Sigma Plot 7.0. Dead meristematic cells were analyzed using the mixed generalized linear model with a Poisson distribution (*p* > 0.05), these analysis were performed using Infostat.

## Results

### Full Inhibition of Primary Root Elongation in Col-0 Seedlings by Ultraviolet-B Requires Direct Exposure of the Roots and It Is a Consequence of Decreased Cell Proliferation in the Meristematic Zone

**FIGURE 1:**
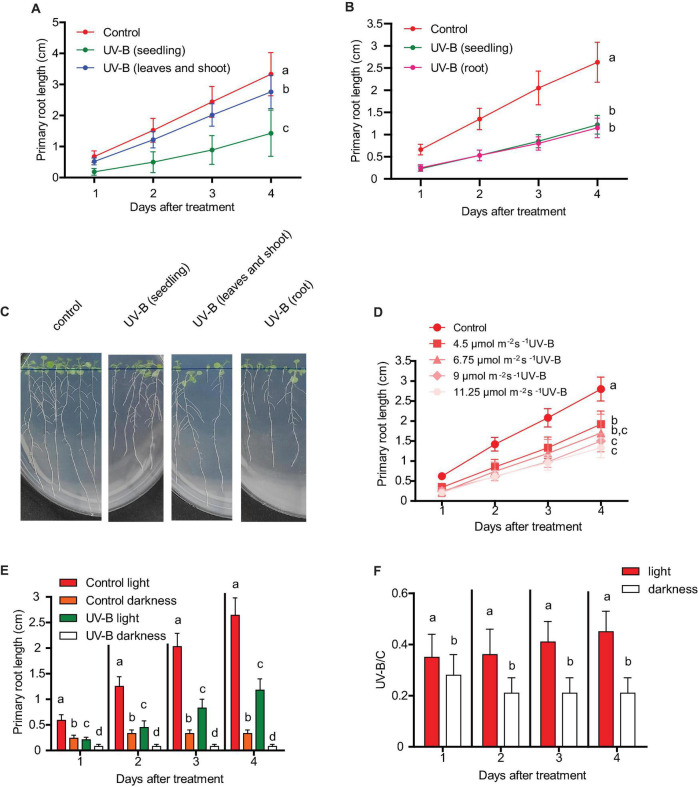
Primary root growth inhibition assays in WT Col-0 after UV-B exposure. **(A)** Graphs of average root lengths of Col-0 seedlings that were grown in the absence of UV-B (control), after UV-B irradiation for 1 h at 9 μmol m^–2^ s^–1^ UV-B of the full seedlings or when only the shoot and leaves were irradiated. **(B)** Graphs of average root lengths of Col-0 seedlings that were grown in the absence of UV-B (control), after UV-B irradiation for 1 h at 9 μmol m^–2^ s^–1^ UV-B of the full seedlings or when only the roots were irradiated. **(C)** Representative pictures of one experiment showing primary roots from WT Col-0 seedlings under the different experimental conditions analyzed in **(A,B)**. **(D)** Fluence response curves of primary root inhibition by UV-B in Col-0 seedlings. Seedlings were irradiated at 4.5, 6.75, 9, or 11.25 μmol m^–2^ s^–1^ UV-B for 1 h. **(E)** Graphs of average root lengths of Col-0 seedlings that were grown in the absence of UV-B (control), after UV-B irradiation for 1 h at 9 μmol m^–2^ s^–1^ UV-B and were then kept under normal photoperiod (light) or in the darkness (dark). **(F)** Ratio between root lengths after UV-B exposure vs. under control conditions shown in **(E)**. Results show the individual values and the average from at least 20 biological replicates ± S.D. from one experiment. Three independent experiments were performed with similar results. Different letters indicate statistically significant differences applying one-way ANOVA (Dunn test, *P* < 0.05).

As described in the section “Introduction,” in Arabidopsis seedlings, UV-B exposure produces an inhibition of primary root elongation. Because Arabidopsis plants have UV-B specific sensing and responsive mechanisms in the roots (Tong et al., 2008; Leasure et al., 2009), and as they also express the UV-B photoreceptor UVR8 in this organ (van Gelderen et al., 2018), we first investigated whether primary root inhibition after UV-B exposure requires full exposure of roots, or if it can also occur when only the shoots and leaves are irradiated, as it generally happens in nature. Figure 1 shows that 1 day after a UV-B treatment, primary roots are significantly shorter than those from non-irradiated plants when they are irradiated for 1 h at an intensity of 9 μmol m^–2^ s^–1^. Interestingly, when only the shoots and leaves were exposed to a similar UV-B treatment, primary roots still showed a significant inhibition of elongation; however, this inhibition was much lower than that measured in fully exposed seedlings (Figures 1A,C). On the contrary, when only roots were UV-B irradiated, the decrease in primary root elongation was similar to that observed in fully irradiated seedlings (Figures 1B,C). Together, these results suggest that although a partial inhibition of primary root elongation by UV-B could be activated from signals from usually directly exposed tissues, such as the shoots and leaves, full inhibition requires direct UV-B exposure of the roots.

Next, we analyzed how primary root inhibition was affected by UV-B treatments at different fluences. Our results show that when seedlings were UV-B irradiated for 1 h at an intensity of 4.5 μmol m^–2^ s^–1^, primary root elongation was less affected than when the experiment was done for the same time using 9 μmol m^–2^ s^–1^ (Figure 1D). However, higher UV-B doses produced a similar inhibition of primary root elongation (Figure 1D), suggesting that at 9 μmol m^–2^ s^–1^ this response gets its maximal inhibitory effect.

The consequence of UV-B exposure in primary root elongation was also studied when irradiated plants were allowed to recover under dark conditions. Figure 1E shows that darkness not only affected primary root elongation in UV-B irradiated plants, but also in control non-irradiated seedlings. However, growth under dark conditions was more affected in UV-B irradiated plants, suggesting that white light is required to improve root elongation in UV-B irradiated seedlings.

**FIGURE 2:**
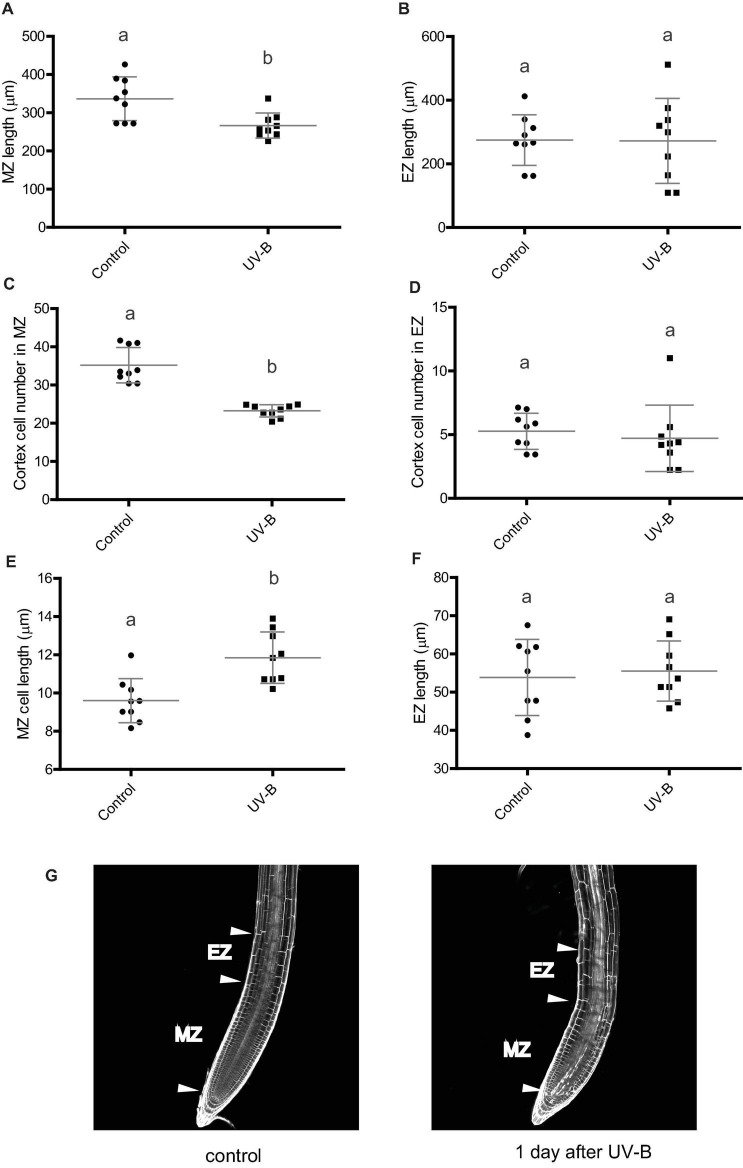
UV-B inhibits cell proliferation in the primary root meristematic zone and does not affect the elongation zone of WT Col-0 seedlings. **(A)** Root meristematic zone length, **(C)** cortex cell number, and **(E)** cortex cell length from control or UV-B treated for 1 h at 9 μmol m^–2^ s^–1^ UV-B WT Col-0 seedlings 1 day after the treatment. **(B)** Root elongation zone length, **(D)** cortex cell number, and **(F)** cortex cell length from UV-B treated or control WT Col-0 seedlings. Results show the individual values and the average from at least eight independent biological replicates ± S.D. from one experiment. Different letters indicate statistically significant differences applying one-way ANOVA (Dunn test, *P* < 0.05). Three independent experiments were performed with similar results. **(G)** Representative images of primary roots of WT Col-0 seedlings 1 day after a UV-B treatment or kept in the absence of UV-B (control).

Arabidopsis primary roots can be divided in three different regions: the meristematic zone, which contains cells that actively divide and elongate; the elongation zone, which includes cells that no longer divide but only elongate (Figure 2G); and the mature zone, containing cells that no longer divide nor elongate (Fiorani and Beemster, 2006). Thus, we investigated whether the decrease in primary root length was a consequence of an inhibition of the growth of either or both the meristematic and the elongation zones, which are those that could affect primary root elongation. One day after a UV-B treatment, while the elongation zone size was not affected by the treatment in WT Col-0 plants, the meristematic zone length was significantly decreased, showing a significant lower number of cortex cells after the treatment (Figures 2A–C). Interestingly, in UV-B irradiated roots, cell length in the meristems was increased compared to that under control conditions (Figure 2E), suggesting that a compensating effect may be taking place to counteract the inhibition of cell proliferation measured. Despite this, this compensation did not completely revert the decrease in the meristem length measured. In contrast, cortex cell number and cortex cell length in the elongation zone was not affected by the UV-B treatment (Figures 2D,F); thus, inhibition of primary root elongation by UV-B is probably due to an inhibition of cell proliferation in the meristem of Col-0 seedlings.

When the effect of a UV-B treatment on the primary root meristematic and elongating zones was analyzed 4 days after the treatment, the results showed that there is still a decrease in the size of the meristem of UV-B treated seedlings, with a lower number of cells with longer sizes, suggesting that the inhibitory effect persists several days after the end of the treatment (Supplementary Figure 1). On the contrary, the elongation zone was not affected 4 days after the treatment, similarly as shown 1 day after the treatment (Supplementary Figure 1).

### Ultraviolet-B Affects Cell Expansion, Cell Division, and Programmed Cell Death in the Primary Root Meristems at Different Root Developmental Stages

**FIGURE 3:**
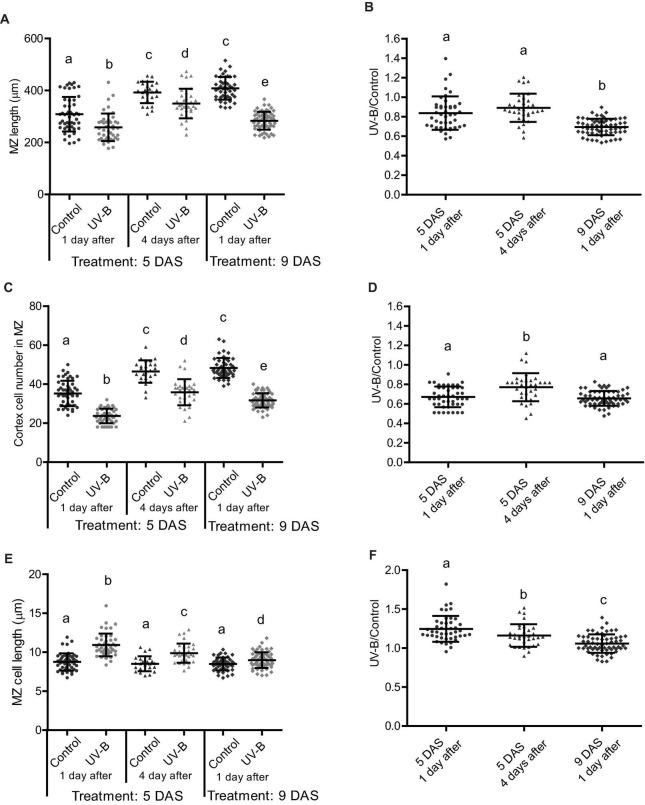
UV-B differently affects cell proliferation in the root meristematic zone of WT Col-0 seedlings at different developmental stages or times after exposure. **(A)** Meristematic zone length, **(C)** cortex cell number, and **(E)** cortex cell length in the primary roots from UV-B treated or control WT Col-0 seedlings 1 or 4 days after the treatment in plants irradiated 5 or 9 days after sowing (DAS). Different letters indicate statistically significant differences applying two-way ANOVA (Tukey’s test, *P* < 0.05). **(B)** Ratio between meristematic zone length, **(D)** cortex cell number, and **(F)** cortex cell length values measured after UV-B exposure vs. those under control conditions in primary roots are shown. Different letters indicate statistically significant differences applying one-way ANOVA (Dunn test, *P* < 0.05). Results show the individual values and the average from at least eight independent biological replicates ± S.D. from one experiment. Three independent experiments were performed with similar results.

Next, we compared how cell expansion and cell elongation in the meristematic zone was affected at 1 and 4 days after recovery from a UV-B treatment, or when irradiation was done at different stages of primary root development. Our results show that 4 days after a UV-B treatment, the primary root meristematic zone is still shorter than that from non-irradiated plants (Figures 3A,B). However, the decrease in cell number is lower than that measured 1 day after the treatment, and this lower decrease in cell proliferation is accompanied with a lower increase in cell expansion (Figures 3C–F), suggesting that inhibition of cell proliferation is recovered over time after the end of the treatment.

In the primary root meristems of Arabidopsis plants, proliferating and expanding cells coexist and are primarily separated in space. At early stages of development, primary root meristem size increases as a consequence of coordinated cell division and elongation, until the meristem size reaches to a maximal size (Fiorani and Beemster, 2006). As shown in Figure 3, the size of the primary root meristems 5 days after sowing in control seedlings not exposed to UV-B is still growing, as 9 days after sowing the size of the meristems is longer; with more cortex cells. Thus, we investigated the effect of UV-B exposure in Arabidopsis seedlings where the meristem has completely expanded, and primary root elongation is mostly achieved by increased number of mature cells (Fiorani and Beemster, 2006). Figure 3B shows that the decrease in meristem size 1 day after the UV-B treatment at 9 DAS was more important than when it was applied 5 DAS. Interestingly, inhibition of cell proliferation 1 day after the treatment was similar when seedlings were irradiated at 5 and 9 DAS (Figures 3C,D). However, the decrease in the meristem size was larger in these plants because at this developmental stage, cell elongation was less increased by UV-B (Figures 3E,F).

**FIGURE 4:**
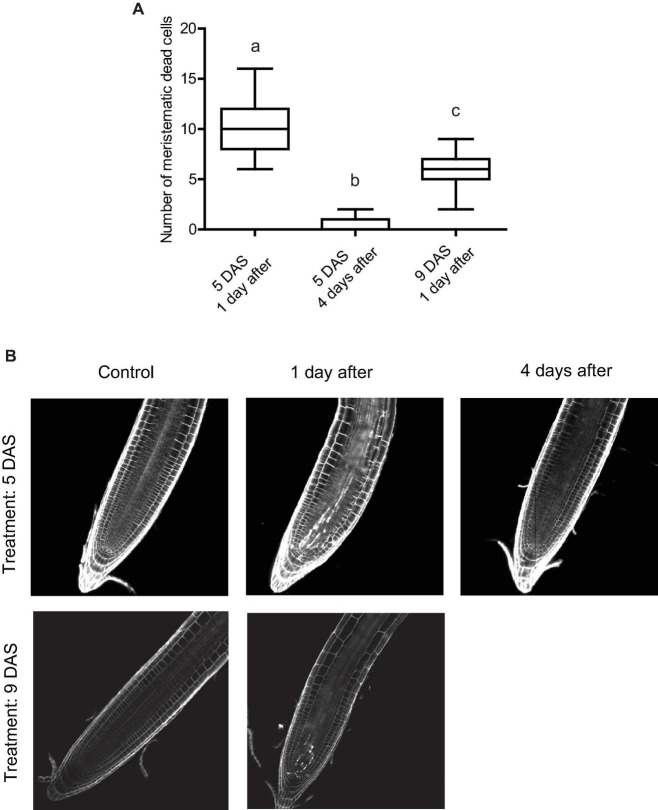
UV-B affects programmed cell death in the root meristematic zone of WT Col-0 seedlings. **(A)** Number of stem cells that are dead after UV-B exposure in WT Col-0 primary root meristems 1 and 4 days after the treatment 5 days after sowing (DAS), or 1 day after the treatment 9 DAS. Results show the individual values and the average from at least 15 independent biological replicates ± S.D. from one experiment. Different letters represent statistically significant differences applying a mixed generalized linear model with a Poisson distribution (*p* > 0.05). Three independent experiments were performed with similar results. **(B)** Representative images of primary roots of WT Col-0 seedlings in which stem cells and adjacent daughter cells were PI staining to count dead stem cells per root 1 or 4 days after UV-B exposure or in control conditions 5 or 9 DAS.

After Arabidopsis seedlings are exposed to UV-B radiation, there is also an activation of programmed cell death (PCD) in the primary root meristematic zone (Furukawa et al., 2010). Dead cells are stained when incubated with propidium iodide (PI), while live cells exclude this compound (Furukawa et al., 2010). Thus, we next analyzed how PCD activation was affected at different times after exposure or when UV-B irradiation was done at different developmental stages. Figure 4 shows that when UV-B irradiation was done when the meristematic zone is still growing (5 DAS), the number of dead cells was significantly higher than when the treatment was done after the meristem has reached its final size. Interestingly, 4 days after UV-B exposure, primary roots from irradiated seedlings at 5 DAS showed a significant reduction of dead cells, as previously reported (Johnson et al., 2018; Maulión et al., 2019).

Together, these results demonstrate that in the primary root meristems, inhibition of cell proliferation, increase in cell expansion and PCD after UV-B exposure take place at different developmental stages and remain several days after the treatment. However, the degree to which these processes are affected depend on the developmental stage of the primary roots and the time after exposure.

### Growth Regulating Factor 3 Regulates Primary Root Elongation, Inhibition of Cell Proliferation and Programmed Cell Death in the Meristematic Zone After Ultraviolet-B Exposure

**FIGURE 5:**
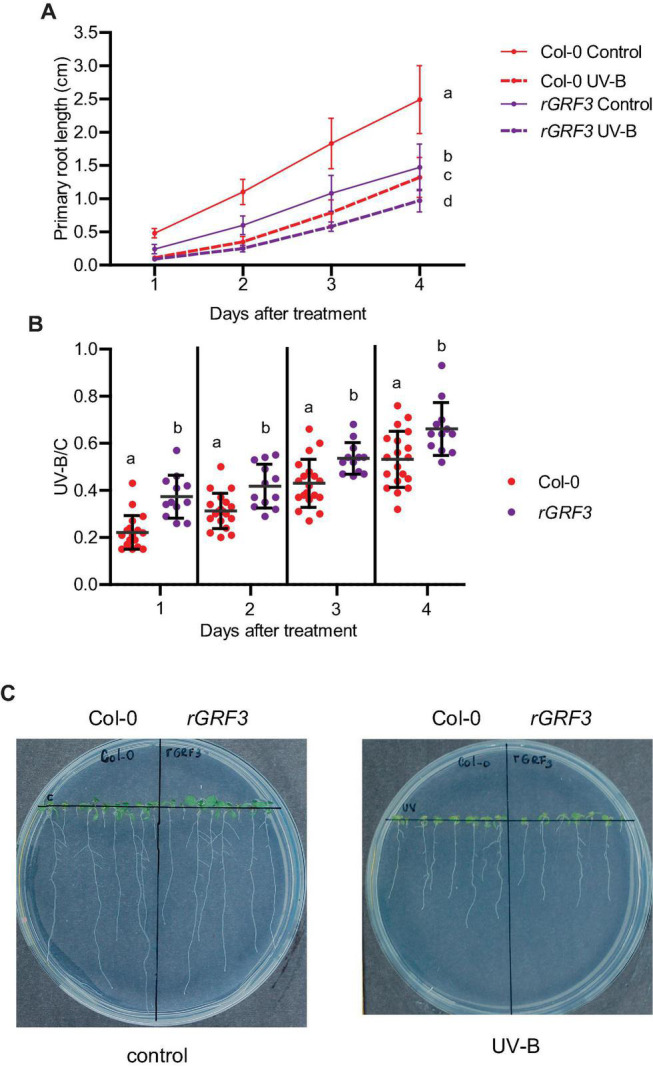
Primary root growth inhibition assays in WT Col-0 and *rGRF3* seedlings after UV-B exposure. **(A)** Graphs of average root lengths of WT Col-0 and *rGRF3* seedlings after UV-B exposure or grown under control conditions without UV-B. **(B)** Ratio between root lengths after UV-B exposure vs. under control conditions. Results show the individual values and the average from at least 20 biological replicates ± S.D. from one experiment. Three independent experiments were performed with similar results. Different letters indicate statistical significant differences applying one-way ANOVA (Dunn test, *P* < 0.05). **(C)** Representative pictures of one experiment showing primary roots from control and UV-B irradiated WT Col-0 and *rGRF3* seedlings.

Previously, we demonstrated that after UV-B exposure, inhibition of leaf growth was mediated by the miR396 and the Growth Regulating Factors (GRFs), including GRF3. Therefore, we investigated if transcription factors from this family also participate in the inhibition of primary root elongation in UV-B irradiated seedlings. For these experiments, we analyzed the effect of UV-B radiation in cell proliferation in transgenic plants expressing a miR396-resistant version of GRF3 under its own promoter (*rGRF3*). This transgene was prepared by introducing synonymous mutations in the miR396 binding site of GRF3 (Casadevall et al., 2013). Primary roots from *rGRF3* plants were shorter than those from WT Col-0 plants when grown under control conditions as previously reported (Rodriguez et al., 2015; Figure 5). After UV-B exposure, *rGRF3* roots were still shorter than WT roots (Figure 5A), but showed a lower inhibition of elongation by UV-B (Figure 5B).

**FIGURE 6:**
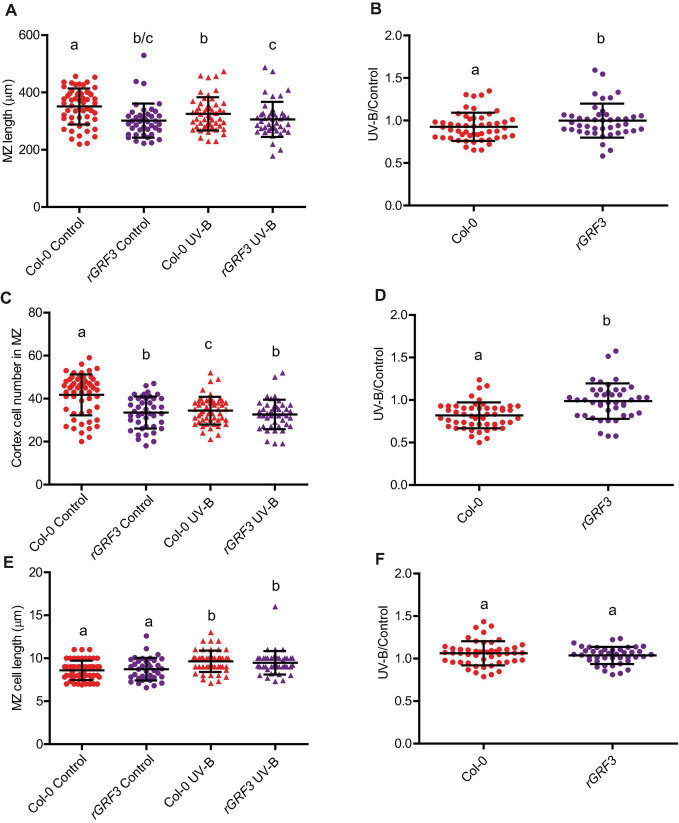
UV-B differently affects cell proliferation in the primary root meristematic zone of WT Col-0 and *rGRF3* seedlings. **(A)** Root meristematic zone length, **(C)** cortex cell number, and **(E)** cortex cell length from UV-B treated or control WT Col-0 and *rGRF3* seedlings 4 days after the treatment. Different letters indicate statistically significant differences applying two-way ANOVA (Tukey’s test, *P* < 0.05). **(B)** Ratio between meristematic zone length, **(D)** cortex cell number, and **(F)** cortex cell area values measured after UV-B exposure vs. those under control conditions in primary roots are shown. Different letters indicate statistically significant differences applying one-way ANOVA (Dunn test, *P* < 0.05). Results show the individual values and the average from at least eight independent biological replicates ± S.D. from one experiment. Three independent experiments were performed with similar results.

**FIGURE 7:**
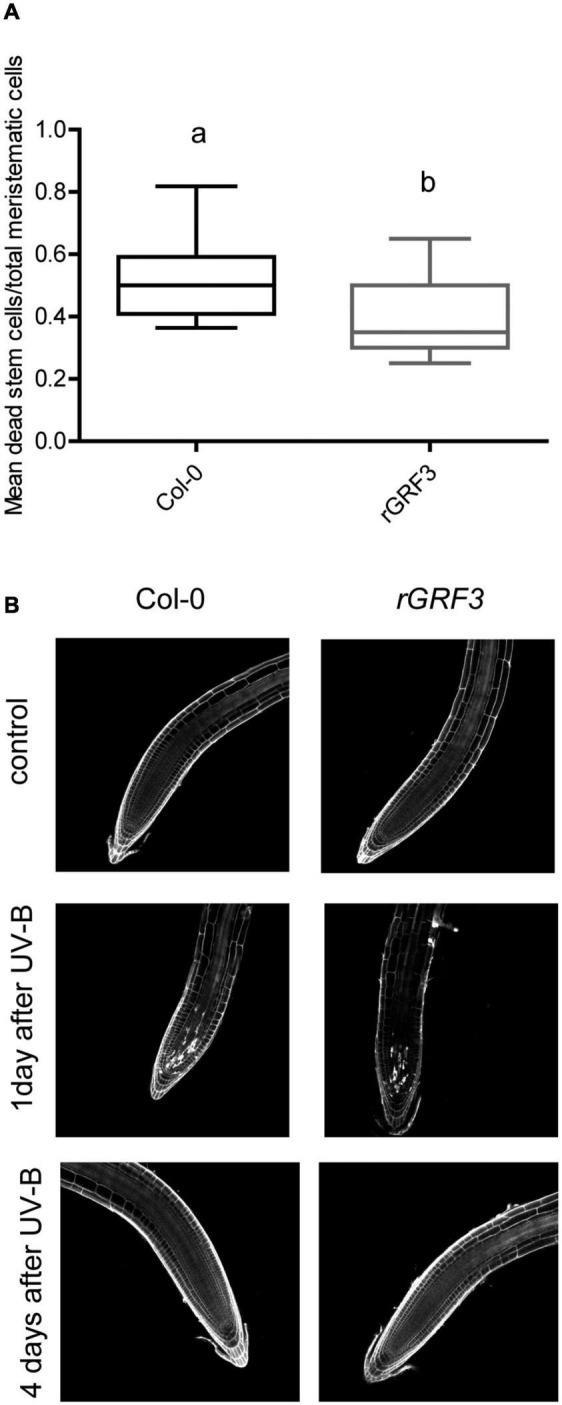
UV-B affects programmed cell death in the root meristematic zone of *rGRF3* seedlings. **(A)** Number of stem cells that are dead 1 day after UV-B exposure in WT Col-0 and *rGRF3* primary root meristems. Results show the individual values and the average from at least 15 independent biological replicates ± S.D. from one experiment. Different letters represent statistically significant differences applying a mixed generalized linear model with a Poisson distribution (*p* > 0.05). Three independent experiments were performed with similar results. **(B)** Representative images of primary roots of WT Col-0 and *rGRF3* seedlings in which stem cells and adjacent daughter cells were PI staining to count dead stem cells per root 1 and 4 days after UV-B exposure or in control conditions without UV-B.

When the effect of UV-B on primary root meristems was compared in WT and *rGRF3* seedlings, the results showed that, in the transgenic plants, inhibition of cell proliferation in the primary roots was reduced compared to WT roots, while cell elongation was not affected (Figure 6). As previously reported, *rGRF3* primary root meristems were shorter with less cells than those from WT seedlings (Rodriguez et al., 2015; Figure 6). Together, our results suggest that similarly as what it was previously reported in proliferating leaves, inhibition of cell proliferation in the roots requires the activity of GRF3. Because *rGRF3* primary root meristems were shorter than WT meristems, the number of dead cells 1 day after the treatment were calculated relative to the cortex cells in this zone, showing that *rGRF3* primary roots had less dead cells after the treatment (Figure 7). This result suggests that GRF3 not only regulates cell proliferation but also PCD after UV-B exposure. Four days after the treatment, both WT and *rGRF3* primary roots showed undetectable levels of dead cells; thus, roots from both lines are similarly recovered in the absence of UV-B (Figure 7B).

### Regulation of Cell Number in the Primary Root Meristems by Ultraviolet-B Is Not Mediated by UVR8, MPK3 or Ataxia Telangiectasia Mutated, but Requires ATM and Rad3-Related and It Is Not Affected in DNA Repair Deficient Mutants

**FIGURE 8:**
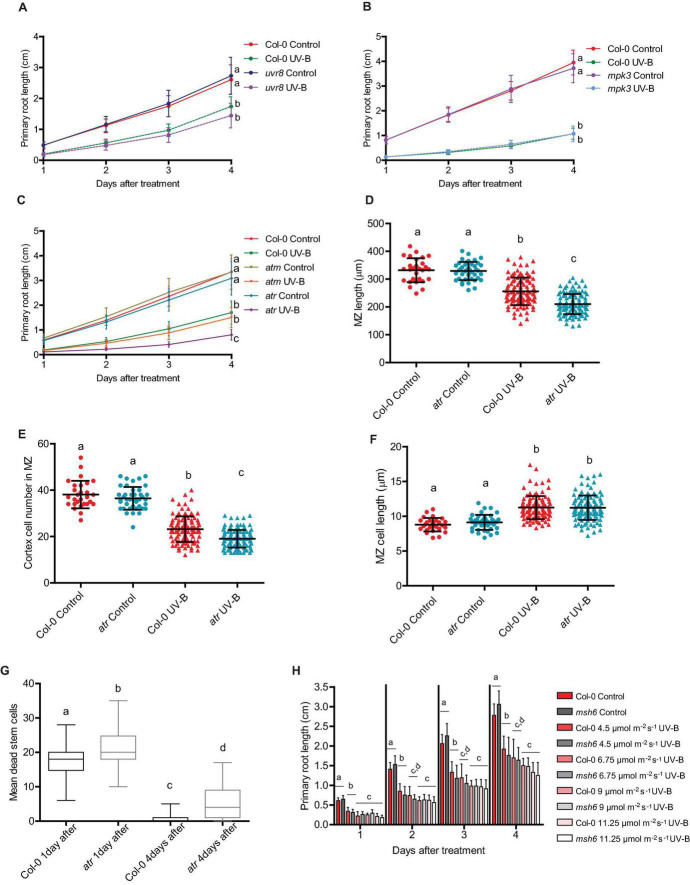
Primary root growth inhibition assays in WT Col-0, *uvr8*, *mpk3*, *msh6*, *atm*, and *atr* seedlings after UV-B exposure. **(A–C)** Graphs of average root lengths in WT Col-0, *uvr8* **(A)**, *mpk3* **(B)**, *atm* and *atr* **(C)** seedlings after UV-B exposure for 1 h at 9 μmol m^–2^ s^–1^ UV-B at or grown under control conditions without UV-B. Results show the individual values and the average from at least 20 biological replicates ± S.D. from one experiment. Three independent experiments were performed with similar results. Different letters indicate statistically significant differences applying two-way ANOVA (Tukey’s test, *P* < 0.05). **(D)** Root meristematic zone length, **(E)** cortex cell number, and **(F)** cortex cell length from UV-B treated or control WT Col-0 and *atr* seedlings 1 day after the treatment. Different letters indicate statistically significant differences applying two-way ANOVA (Tukey’s test, *P* < 0.05). **(G)** Number of stem cells that are dead 1 and 4 days after UV-B exposure in WT Col-0 and *atr* primary root meristems. Different letters represent statistically significant differences applying a mixed generalized linear model with a Poisson distribution (*p* > 0.05). Results show the individual values and the average from at least 15 independent biological replicates ± S.D. from one experiment. Three independent experiments were performed with similar results. **(H)** Fluence response curves of primary root inhibition by UV-B in Col-0 and *msh6* seedlings. Seedlings were irradiated at 4.5, 6.75, 9, or 11.25 μmol m^–2^ s^–1^ UV-B for 1 h. Results show the individual values and the average from at least 20 biological replicates ± S.D. from one experiment. Three independent experiments were performed with similar results. Different letters indicate statistically significant differences applying two-way ANOVA (Tukey’s test, *P* < 0.05).

As described in the section “Introduction,” UV-B responses activated by low-fluence UV-B radiation are mostly regulated by the UV-B specific UVR8 photoreceptor (Rizzini et al., 2011). Nevertheless, some responses to higher UV-B doses are independent of UVR8 and require the activation of different pathways that involve the activity of several protein kinases, for example MPK3, ATM, or/and ATR (Ulm, 2003). Previously, we showed that inhibition of cell division by UV-B in proliferating leaves of Arabidopsis by miR396 and GRFs depended on the activation of MPK3 but was independent of UVR8, MPK6, ATM, and ATR (Casadevall et al., 2013). Thus, we further investigated if a similar regulation occurs in the primary roots. As shown in Figure 8A, *uvr8* mutants showed a similar inhibition of root elongation after UV-B exposure; with a similar reduction of the meristematic zone length after the treatment (Supplementary Figure 2). The decrease in the number of cortex cells and increase in cell length after the treatment was also similar as that in WT roots, suggesting that this response to UV-B is independent of the photoreceptor. Interestingly, a similar response was measured when experiments were done using *mpk3* mutants (Figure 8B and Supplementary Figure 3). This demonstrates that inhibition of cell proliferation in the roots by UV-B is activated by a different pathway as that occurring in leaves, independently of MPK3.

On the other hand, while primary roots from *atm* mutants also showed a similar inhibition of elongation after a UV-B treatment by UV-B as WT plants, elongation of *atr* primary roots was more inhibited than WT or *atm* roots (Figure 8C). Under control conditions, the meristematic zone length from *atr* roots was similar to that from WT roots, with a similar number of cells of similar length (Figures 8D–F). However, 1 day after a UV-B treatment, the meristem size was significantly more shortened, showing a larger decrease in the number of cortex cells than that in the meristematic zone of WT Col-0 seedlings, but a similar increase in cell length (Figures 8D–F). Moreover, *atr* mutants accumulated more dead cells after UV-B exposure, both 1 and 4 days after the treatment (Figure 8G), while neither *uvr8* nor *mpk3* mutants showed differences in the number of dead cells compared to WT seedlings (Supplementary Figures 2D,E, 3D). Therefore, while inhibition of cell proliferation by UV-B in the leaves and in the primary roots require the activity of GRF3, MPK3 only regulates cell proliferation in the leaves while ATR is required for proper inhibition of cell division in the roots.

In previous studies, mutants in DNA repair enzymes involved in Nucleotide Excision Repair showed a more important decrease in hypocotyl elongation by UV-B than WT seedlings (Gardner et al., 2009; Biever et al., 2014). These results suggested that direct damage to DNA may regulate photomorphogenic responses. In our lab, we previously demonstrated that Arabidopsis mutants in *MSH2* and *MSH6*, encoding two proteins that participate in the Mismatch Repair DNA pathway, were deficient in DNA repair after UV-B exposure (Lario et al., 2011). Thus, we investigated whether *msh6* primary roots were more affected by UV-B than WT roots. Our results showed that elongation of primary roots from *msh6* mutants was similarly inhibited by UV-B radiation as those from WT seedlings, and this was also true when irradiation was done using different UV-B fluences (Figure 8H). Interestingly, elongation of *msh6* primary roots was similarly inhibited by UV-B exposure when seedlings were allowed to recover under normal photoperiod in the presence of white light that allow photorepair of damaged DNA, or under darkness (Supplementary Figure 4). These results demonstrate that although *msh6* mutants accumulate more DNA damage after UV-B exposure than WT plants, they show a similar inhibition of root growth at similar UV-B fluences and also under conditions that do not allow photorepair, suggesting that levels of DNA damage accumulated in WT plants after UV-B exposure are sufficient to activate inhibition of cell proliferation in the primary root meristems.

### Ultraviolet-B Inhibition of Root Elongation Differ in Arabidopsis Ecotypes and Accessions

**FIGURE 9:**
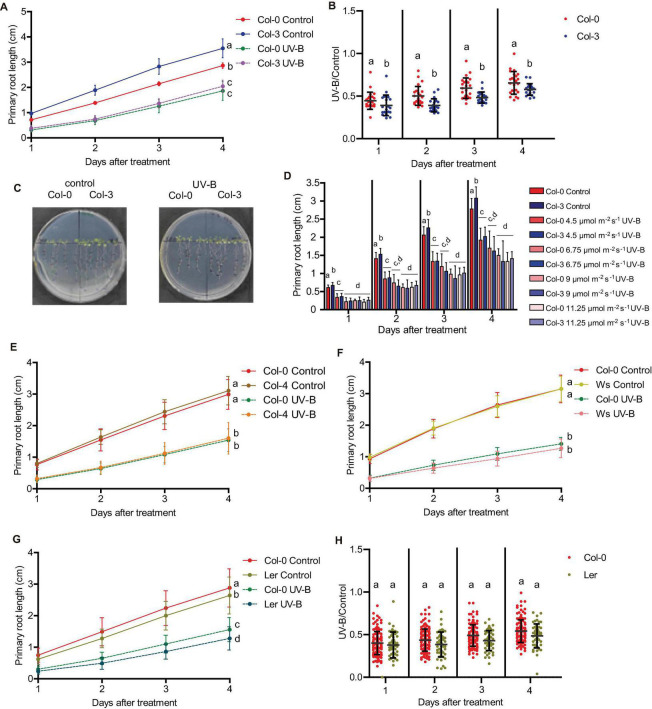
Primary root growth inhibition assays in WT Col-0, Col-3, Col-4, Ws, and Ler seedlings after UV-B exposure. **(A)** Graphs of average root lengths in WT Col-0 and Col-3 seedlings after UV-B exposure for 1 h at 9 μmol m^–2^ s^–1^ UV-B or grown under control conditions without UV-B. **(B)** Ratio between root lengths after UV-B exposure vs. under control conditions. **(C)** Representative pictures of one experiment showing primary roots from control and UV-B irradiated WT Col-0 and Col-3 seedlings. **(D)** Fluence response curves of primary root inhibition by UV-B in Col-0 and Col-3 seedlings. Seedlings were irradiated at 4.5, 6.75, 9, or 11.25 μmol m^–2^ s^–1^ UV-B for 1 h. **(E–G)** Graphs of average root lengths in WT Col-0 and Col-4 **(E)**, Ws **(F)**, and Ler **(G)** seedlings after UV-B exposure or grown under control conditions without UV-B. Results show the individual values and the average from at least 20 biological replicates ± S.D. from one experiment. **(H)** Ratio between Col-0 and Ler root lengths after UV-B exposure vs. under control conditions. Three independent experiments were performed with similar results. Different letters indicate statistically significant differences applying one-way ANOVA (Dunn test, *P* < 0.05).

Finally, we analyzed weather the different primary root phenotypes in Col-0 seedlings after UV-B exposure were also observed in other Arabidopsis accessions and ecotypes. When primary root elongation was compared in Col-0, Col-3, Col-4, Ws, and Ler seedlings after UV-B exposure, all lines showed a significant inhibition of growth (Figure 9). However, while primary roots from Col-3 were longer than those from Col-0 seedlings, after UV-B exposure at different fluences, primary roots from both accessions showed a similar length (Figures 9A–D). It is interesting to note that higher inhibition of primary root elongation in Col-3 seedlings occurs even at low UV-B fluences, and the inhibitory effect is similar under all UV-B conditions investigated (Figure 9D). Thus, elongation of Col-3 primary roots is more affected by UV-B. In contrast; primary roots from all other accessions/ecotypes analyzed showed a similar inhibition of elongation after a UV-B treatment as Col-0 (Figures 9E–H).

**FIGURE 10:**
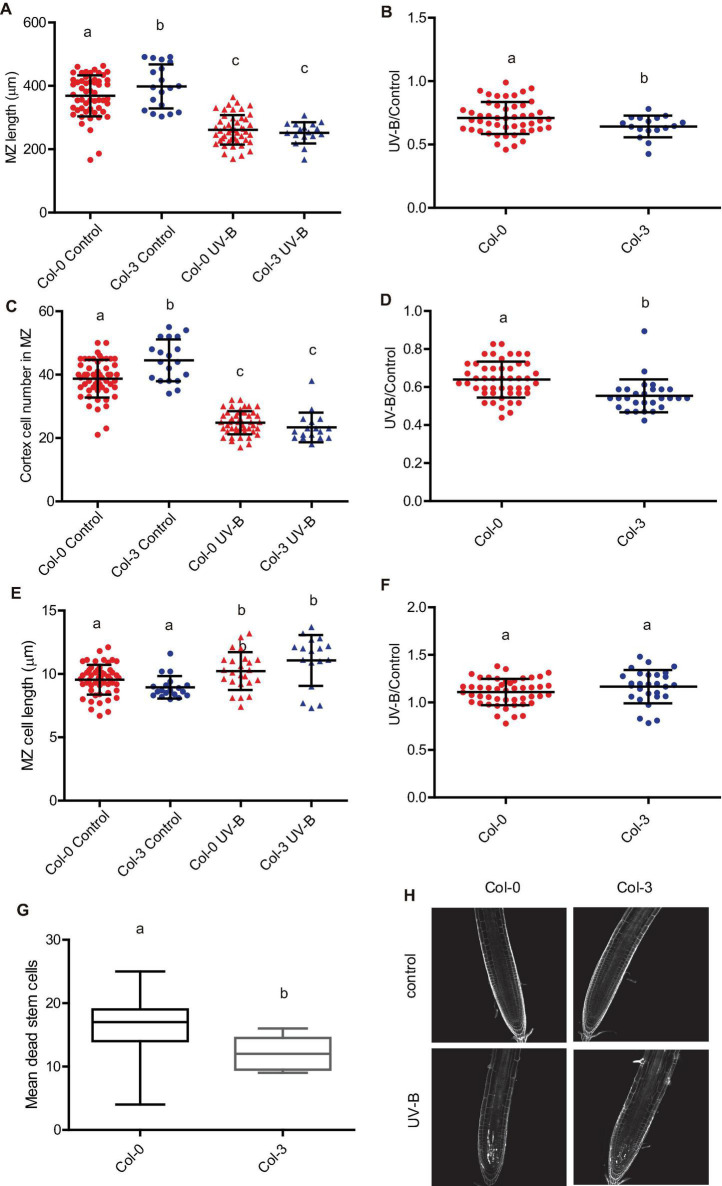
UV-B differently affects cell proliferation and PCD in the root meristematic zone of WT Col-0 and Col-3 seedlings. **(A)** Root meristematic zone length, **(C)** cortex cell number, and **(E)** cortex cell length from UV-B treated or control WT Col-0 and Col-3 seedlings 1 day after the treatment. Different letters indicate statistical significant differences applying two-way ANOVA (Tukey’s test, *P* < 0.05). **(B)** Ratio between meristematic zone length, **(D)** cortex cell number, and **(F)** cortex cell area values measured after UV-B exposure vs. those under control conditions in primary roots are shown. Different letters indicate statistically significant differences applying one-way ANOVA (Dunn test, *P* < 0.05). Results show the individual values and the average from at least eight independent biological replicates ± S.D. from one experiment. **(G)** Number of stem cells that are dead 1 day after UV-B exposure in WT Col-0 and Col-3 primary root meristems. Different letters represent statistically significant differences applying a mixed generalized linear model with a Poisson distribution (*p* > 0.05). Results show the individual values and the average from at least 15 independent biological replicates ± S.D. from one experiment. Three independent experiments were performed with similar results. **(H)** Representative images of primary roots of WT Col-0 and Col-3 seedlings in which stem cells and adjacent daughter cells were PI staining to count dead stem cells per root 1 day after UV-B exposure or in control conditions without UV-B.

In addition, all accessions except Col-3 showed a similar inhibition of the meristematic zone length after UV-B exposure, with a similar decrease in cell proliferation after the treatment (Figure 10 and Supplementary Figure 5). In contrast, Col-3 meristematic zone was longer than that from Col-0 roots, but after the treatment, meristems from both accessions showed a similar length (Figure 10A). The higher decrease in the meristem size in Col-3 was a consequence of a higher inhibition of cell proliferation after the treatment (Figures 10C,D), while the increase in cell length was similar in both accessions (Figures 10E,F). Together, Col-3 primary roots are more sensitive to UV-B than those from the other accessions and ecotypes analyzed.

Interestingly, when PCD was studied 1 day after a UV-B exposure, not only Col-3 but also Col-4 primary roots meristems showed decreased number of dead cells compared to Col-0, while Ler and Ws roots showed similar PCD as Col-0 (Figures 10G,H and Supplementary Figure 5). Interestingly, all accessions showed similar DNA damage accumulation after UV-B exposure (Supplementary Figures 5J,K). These results demonstrate that while primary roots from all accessions analyzed are affected by a UV-B, there is natural variation in the responses measured.

## Discussion

In the primary roots of *Arabidopsis thaliana* plants, the meristematic zone located in the root tips contains cells that actively divide and elongate; while the elongation zone includes cells that no longer divide but only elongate, and the mature zone has cells that no longer divide nor elongate (Fiorani and Beemster, 2006). Cells are generally organized in parallel files along which one-dimensional developmental gradients are present. Therefore, primary root elongation is mostly a consequence of cell proliferation in the meristematic zone, and cell expansion in the meristematic and elongation zones. In this work, we investigated the effect of a single UV-B radiation treatment on the primary roots of Arabidopsis seedlings, and we demonstrated that UV-B exposure can inhibit its elongation by decreasing cell proliferation in the meristematic zone. We also here show that the decrease in cell proliferation is accompanied with an increase in cell elongation; thus, a compensating effect occurs to balance the inhibition of cell proliferation measured. However, the effect of UV-B on cell proliferation is more important than that on cell expansion, and the consequence of this is that the meristem size of irradiated primary roots is smaller than that from control roots (Figure 2). In contrast, cortex cell number and cortex cell length in the elongation zone was not affected by the UV-B treatment (Figure 2); thus, inhibition of primary root elongation by UV-B is probably mostly due to an inhibition of cell proliferation in the meristematic zone of Col-0 seedlings. Interestingly, when we previously studied the effect of a UV-B treatment on Arabidopsis leaves, which were grown at similar intensities as those used in this study, our results showed that in proliferating leaves, only cell division was affected, while cell size was not modified (Casadevall et al., 2013). Moreover, similar experiments using maize plants also demonstrated that cell number in the meristematic zone of growing leaves was decreased by UV-B but not cell size, and this decrease in cell number caused a decrease in the meristematic zone size (Fina et al., 2017). Maize is a monocot plant, and elongation of growing leaves occurs in a similar way as primary root elongation in Arabidopsis (Fiorani and Beemster, 2006; Fina et al., 2017). Our previous study using maize leaves showed that UV-B affected the meristematic zone size without changing the elongation zone, similarly as what is now described for primary roots from *Arabidopsis thaliana* seedlings (Fina et al., 2017; Figure 2). Together, these results demonstrate that exposure to UV-B at our experimental conditions affects cell proliferation in leaves and roots but only cell expansion in the root meristems. The increase in cell size could be a consequence of changes in the cell wall structure or composition occurring after exposure, as previously described in the leaf cell walls of mutants with altered sensitivity to this radiation (Maulión et al., 2019), to changes in endoreduplication levels in the cells, which are sometimes measured after UV-B exposure in leaf cells (Radziejwoski et al., 2011), or to other UV-B modulated mechanisms that could specifically affect cell size in the roots not yet characterized.

On the other hand, our results also show that UV-B inhibits cell division in the primary root meristems at different developmental stages, but the degree of inhibition and the effect in cell elongation varies according to the development stage of the meristematic zone. Interestingly, 4 days after irradiation, meristems from UV-B exposed plants were still smaller than those control plants, with less but longer cells (Figure 3), demonstrating that the developmental consequences of a UV-B treatment are maintained several days in the absence of the stress. On the contrary, while the number of dead cells in the meristems 1 day after a UV-B treatment was substantial, 4 days after the treatment most roots showed a significant recovery with very low number of dead cells (Figure 4). Therefore, in the root meristematic zone there is a faster recovery from PCD than from developmental changes that occur after UV-B exposure. In agreement with the results presented here, Johnson et al. (2018) showed that, after DNA damage occurs, programmed cell death in the root meristematic cells triggers its regeneration and enables growth recovery. This process is regulated by the activity of the transcription factor SUPPRESSOR OF GAMMA RESPONSE 1 (SOG1), which activates DNA damage-induced programmed cell death. In this way, proper activation and then recovery of PCD after DNA damage from UV-B is required to undergo normal cell division rates. Thus, and as also proposed by Johnson et al. (2018), recovery from programmed cell death could be a signal employed by plants to restore growth after repair of DNA damage following UV-B exposure. Previously, Gardner et al. (2009) showed that, similarly as what we here report occurs in Arabidopsis seedlings primary roots, UV-B exposure induces growth inhibition of etiolated seedlings. As a consequence, inhibition of hypocotyl elongation was observed, which depended on photon fluence. In our experiments, the degree of primary root inhibition also depended on UV-B fluence. Mutants in DNA repair enzymes involved in Nucleotide Excision Repair showed a more important decrease in hypocotyl elongation by UV-B than WT seedlings, suggesting that direct damage to DNA may regulate photomorphogenic responses, probably repressing cell cycle progression (Gardner et al., 2009; Biever et al., 2014). In contrast, in our experiments, *msh6* mutants, which are deficient in DNA repair after UV-B exposure (Lario et al., 2011) showed a similar inhibition of primary root elongation as WT seedlings at all UV-B fluences studied, and even under dark conditions that do not allow photorepair of the DNA by photolyases. This suggests that levels of DNA damage accumulated in WT plants after UV-B exposure are sufficient to inhibit cell proliferation in the primary root meristems. Thus, inhibition of cell proliferation caused by UV-B are probably a consequence of an activation of the DNA damage response that could occur at different fluences in the different organs. It is important to note that, as reviewed by Biever and Gardner (2016), it is evident that some photomorphogenic responses, such as the inhibition of hypocotyl and primary root elongation in Arabidopsis seedlings, respond to UV-B-induced DNA damage and do not require the activation of the UVR8 photoreceptor, and this could also be dependent on the UV-B fluence used during the irradiation treatments.

Interestingly, while complete inhibition of the primary root elongation by UV-B requires full exposure of this organ, irradiation of the leaves and shoots provokes a partial but significant decrease in the root length (Figure 1). Thus, it is possible that signals from these tissues could be transmitted to the roots. Previously, we demonstrated that maize plants that were irradiated with UV-B lamps at similar intensities used in the experiments described here showed transcriptome changes in the roots, which were not directly exposed to UV-B (Casati and Walbot, 2004). In this previous study, transcriptome changes were also measured in different types of maize shielded tissues besides roots, such as immature ears and shielded leaves, and these different tissues all displayed altered transcriptome profiles after exposure of the plant to UV-B (Casati and Walbot, 2004). Thus, some signal(s) must be transmitted from irradiated to shielded tissues in different plants species, and in Arabidopsis, these signals could regulate root growth in the absence of direct exposure.

Our data provides evidence that primary root inhibition by UV-B is at least in part regulated by GRF3, a transcription factor from the Growth regulating family that is a target of the microRNA miR396. In the experiments presented here, the decrease in cortex cell number after a UV-B exposure in the primary root meristems was partially compensated by an increase in cell length, but the regulation of cell elongation was independent of GRF3, as cells from *rGRF3* roots were similarly elongated as those from WT after a treatment. The role of GRF3 and other GRFs in root growth has been previously described (Rodriguez et al., 2015; Ercoli et al., 2018). While decreased levels of GRFs and their co-activators GRF-INTERACTING FACTORS (GIFs) leads to an increased size of the primary root meristem, a reduction in miR396 levels, *rGRF2* or *3* expression or *GIF* overexpression decrease the meristem size. In agreement, primary roots from *rGRF3* seedlings in our experiments were also shorter, with smaller meristematic zones (Figures 5, 6). In the roots, GRFs and GIFs regulate *PLETHORA* genes, which are transcription factors that control root growth (Rodriguez et al., 2015; Ercoli et al., 2018). GRF2/3 are expressed in actively dividing cells in the root meristems, and together with GIFs directly decrease *PLETHORA* genes transcription (Rodriguez et al., 2015; Ercoli et al., 2018). Thus, the regulation of cell proliferation by GRFs after UV-B exposure in the roots may also involve the participation of PLETHORA transcription factors.

In Arabidopsis and maize, inhibition of cell proliferation in the leaves by UV-B is regulated by the microRNA miR396 (Casadevall et al., 2013; Fina et al., 2017). miR396 also regulates primary root elongation after UV-B exposure in Arabidopsis (Gómez et al., 2019). Interestingly, it was recently reported that a different microRNA, miR5642, has a potential role regulating UV-B responses during early development in Arabidopsis seedlings (Dukowic-Schulze et al., 2021). This microRNA seems to participate in hypocotyl growth inhibition after UV-B exposure, regulating transcript levels of putative target mRNAs. Both miR396 and miR5642 participate in DNA damage responses activated by UV-B, in particular regulating cell cycle arrest (Casadevall et al., 2013; Dukowic-Schulze et al., 2021). Thus, it would be interesting to investigate whether these two miRNAs participate in a common pathway in response to UV-B.

We have previously showed that, in the leaves, inhibition of cell division by UV-B required the presence of MPK3 (Casadevall et al., 2013). This MPK, together with MPK6 and their phosphatase MKP1, participates in UV-B stress responses in Arabidopsis leaves (González Besteiro and Ulm, 2013). In this species, both MPK3 and MPK6 are activated by UV-B–induced DNA damage. Our results show that, in the primary root meristems, inhibition of cell proliferation by UV-B is independent of MPK3 (Figure 8 and Supplementary Figure 3). However, roots from *atr* seedlings are highly sensitive to a UV-B treatment, showing a higher inhibition of root elongation with a larger decrease in the meristem size of irradiated seedlings (Figure 8). In the leaves of Arabidopsis plants exposed to UV-B, *atr* mutants did not show differences in cell proliferation with WT leaves (Casadevall et al., 2013). ATR participates in a different UV-B activated pathway from MPK3 and MPK6, which seems to be mainly present in the roots (González Besteiro and Ulm, 2013). Thus, the results presented here demonstrate that while GRF3 regulates cell proliferation under UV-B conditions both in the leaves and roots, MPK3 is only required for this regulation in the leaves; and a deficiency in ATR expression significantly affects cell division in UV-B exposed roots. Interestingly, *rGRF3* roots showed a lower number of dead cells after UV-B than WT roots (Figure 7), suggesting the GRF3 not only regulates cell proliferation but also other DNA damage responses. Finally, we here also demonstrate that, in our experimental conditions, inhibition of cell proliferation in the roots and leaves after UV-B exposure is independent of the activity of the UV-B photoreceptor UVR8 and the protein kinase ATM (Casadevall et al., 2013; Figure 8 and Supplementary Figure 2). Interestingly, inhibition of hypocotyl elongation by UV-B was previously reported to be independent of the presence of any of the known photoreceptors including UVR8 (Gardner et al., 2009; Biever et al., 2014); which is in agreement with our results. Thus, it is possible that inhibition of hypocotyl and primary root elongation may share at least some components of a common pathway.

Last, we investigated the effect of UV-B exposure in the roots of different Arabidopsis accessions and ecotypes. Our results demonstrate that despite there are differences in the responses to UV-B analyzed between some accessions; all genotypes responded to the treatment, showing an inhibition of cell proliferation in the root meristems that produced, at least in part, an inhibition in primary root elongation. Moreover, UV-B exposure provoked an induction of PCD in the primary root meristems of all plants studied, and they all showed a similar accumulation of DNA damage after UV-B exposure. These data demonstrate that primary roots from all accessions analyzed are affected by UV-B; however, there is natural variation in the UV-B responses in some ecotypes.

## Conclusion

We here show that inhibition of primary root elongation is a consequence of an inhibition of cell proliferation in the meristematic zone of the primary roots that is regulated by GRF3, while the elongation zone size is not affected by the treatment. The decrease in cell number after UV-B exposure is partially compensated by an increase in cell length in the root meristem; however, this compensation is not enough to maintain the meristem size of control roots grown in the absence of UV-B. Our data also demonstrates that there is natural variation in the root responses to UV-B.

## Data Availability Statement

The original contributions presented in the study are included in the article/Supplementary Material, further inquiries can be directed to the corresponding author.

## Author Contributions

MS and PC conceived and designed the study. MS, LS, and MG performed the experiments. MS, LS, MG, and PC analyzed the data. PC wrote the manuscript. All authors contributed to the article and approved the submitted version.

## Conflict of Interest

The authors declare that the research was conducted in the absence of any commercial or financial relationships that could be construed as a potential conflict of interest.

## Publisher’s Note

All claims expressed in this article are solely those of the authors and do not necessarily represent those of their affiliated organizations, or those of the publisher, the editors and the reviewers. Any product that may be evaluated in this article, or claim that may be made by its manufacturer, is not guaranteed or endorsed by the publisher.
