# Supplementary material for: Ultraviolet-B Radiation Represses Primary Root Elongation by Inhibiting Cell Proliferation in the Meristematic Zone of Arabidopsis Seedlings
Source: Front Plant Sci. 2022 Mar 24;13:829336. doi: 10.3389/fpls.2022.829336 (PMC8988989; doi:10.3389/fpls.2022.829336)
Supplement: Supplementary file 1 [file Data_Sheet_1.PDF]

Supplementary Material

Supplementary Figure legends

SUPPLEMENTARY FIGURE S1 UV-B inhibits cell proliferation in the primary root meristematic zone and does not affect the elongation zone of WT Col-0 seedlings 4 days after the treatment. **(A)** Root meristematic zone length, **(C)** cortex cell number and **(E)** cortex cell length from control or UV-B treated WT Col-0 seedlings 4 days after the treatment. **(B)** Root elongation zone length, **(D)** cortex cell number, and **(F)** cortex cell length from UV-B treated or control WT Col-0 seedlings. Results show the individual values and the average from at least 8 independent biological replicates ± S.D from one experiment. Different letters indicate statistically significant differences applying one-way ANOVA (Dunn test, $P < 0.05$). Three independent experiments were performed with similar results. **(G)** Representative images of primary roots of WT Col-0 seedlings 4 days after a UV-B treatment or kept in the absence of UV-B (control).

SUPPLEMENTARY FIGURE S2 UV-B similarly inhibits cell proliferation in the primary root meristematic zone of WT Col-0 and *uvr8* seedlings. **(A)** Root meristematic zone length, **(B)** cortex cell number and **(C)** cortex cell length from control or UV-B treated WT Col-0 and *uvr8* seedlings 1 day after the treatment. Different letters indicate statistically significant differences applying two-way ANOVA (Tuckey test, $P < 0.05$). Results show the individual values and the average from at least 8 independent biological replicates ± S.D from one experiment. **(D)** Number of stem cells that are dead 1 day after UV-B exposure in WT Col-0 and *uvr8* primary root meristems. Different letters represent statistically significant differences applying a mixed generalized linear model with a Poisson distribution ($p > 0.05$). Results show the individual values and the average from at least 15 independent biological replicates ± S.D from one experiment. Three independent experiments were performed with similar results. **(G)** Representative images of primary roots of WT Col-0 and *uvr8* seedlings one day after a UV-B treatment or kept in the absence of UV-B (control).

SUPPLEMENTARY FIGURE S3 UV-B similarly inhibits cell proliferation in the primary root meristematic zone of WT Col-0 and *mpk3* seedlings. **(A)** Root meristematic zone length, **(C)** cortex cell number and **(E)** cortex cell length from control or UV-B treated WT Col-0 and *mpk3* seedlings 1 day after the treatment. Different letters indicate statistically significant differences applying two-way ANOVA (Tuckey test, $P < 0.05$). **(B)** Ratio between meristematic zone length, **(D)** cortex cell number, and **(F)** cortex cell area values measured after UV-B exposure vs those under control conditions in primary roots are shown. Different letters indicate statistically significant differences applying one-way ANOVA (Dunn test, $P < 0.05$). Results show the individual values and the average from at least 8 independent biological replicates ± S.D from one experiment. **(G)** Number of stem cells that are dead 1 day after UV-B exposure in WT Col-0 and *mpk3* primary root meristems. Different letters represent statistically significant differences applying a mixed generalized linear model with a Poisson distribution ($p > 0.05$). Results show the individual values and the average from at least 15 independent biological replicates ± S.D from one experiment. Three independent experiments were performed with similar results.

SUPPLEMENTARY FIGURE S4 UV-B similarly inhibits primary root elongation in WT Col-0 and msh6 seedlings recovered under white light or under dark conditions. Graphs of average root lengths

of Col-0 and *msh6* seedlings that were grown in the absence of UV-B (control), after UV-B irradiation for 1h at 9 µmol m^{-2} s^{-1} UV-B and were then kept under normal photoperiod (light) or in the darkness (dark). Results show the individual values and the average from at least 20 biological replicates ± S.D from one experiment. Three independent experiments were performed with similar results. Different letters indicate statistically significant differences applying one-way ANOVA (Dunn test, $P < 0.05$).

SUPPLEMENTARY FIGURE S5 UV-B similarly inhibits cell proliferation but differently affect PCD in the primary root meristematic zone of WT Col-0, Col-4, Ws and Ler seedlings. **(A, D, G)** Root meristematic zone length and **(B, E, H)** cortex cell number from control or UV-B treated WT Col-0, Col-4 **(A, B)**, Ws **(D, E)** and Ler **(G, H)** seedlings 1 day after the treatment. Different letters indicate statistically significant differences applying two-way ANOVA (Tuckey test, $P < 0.05$). Results show the individual values and the average from at least 8 independent biological replicates ± S.D from one experiment. **(C, F, I)** Number of stem cells that are dead 1 day after UV-B exposure in WT Col-0, Col-4 **(C)**, Ws **(F)** and Ler **(I)** primary root meristems. Different letters represent statistically significant differences applying a mixed generalized linear model with a Poisson distribution ($p > 0.05$). Results show the individual values and the average from at least 15 independent biological replicates ± S.D from one experiment. **(J, K)** Relative CPD levels in the DNA of seedlings under control conditions in the absence of UV-B and immediately after a 1h **(J)** or 4-h **(K)** UV-B treatment. **(J)** Experiments were done using WT Col-0 and Col-3 seedlings. **(K)** Experiments were done using WT Col-0, Col-3, Col-4, Ws and Ler seedlings. Results represent averages ± S.D. of six independent biological replicates. Different letters indicate statistically significant differences applying two-way ANOVA (Tuckey test, $P < 0.05$).
