# Supplementary material for: Ultraviolet-B Radiation Represses Primary Root Elongation by Inhibiting Cell Proliferation in the Meristematic Zone of Arabidopsis Seedlings
Source: Front Plant Sci. 2022 Mar 24;13:829336. doi: 10.3389/fpls.2022.829336 (PMC8988989; doi:10.3389/fpls.2022.829336)

SUPPLEMENTARY FIGURE S1 UV-B inhibits cell proliferation in the primary root meristematic zone and does not affect the elongation zone of WT Col-0 seedlings 4 days after the treatment.

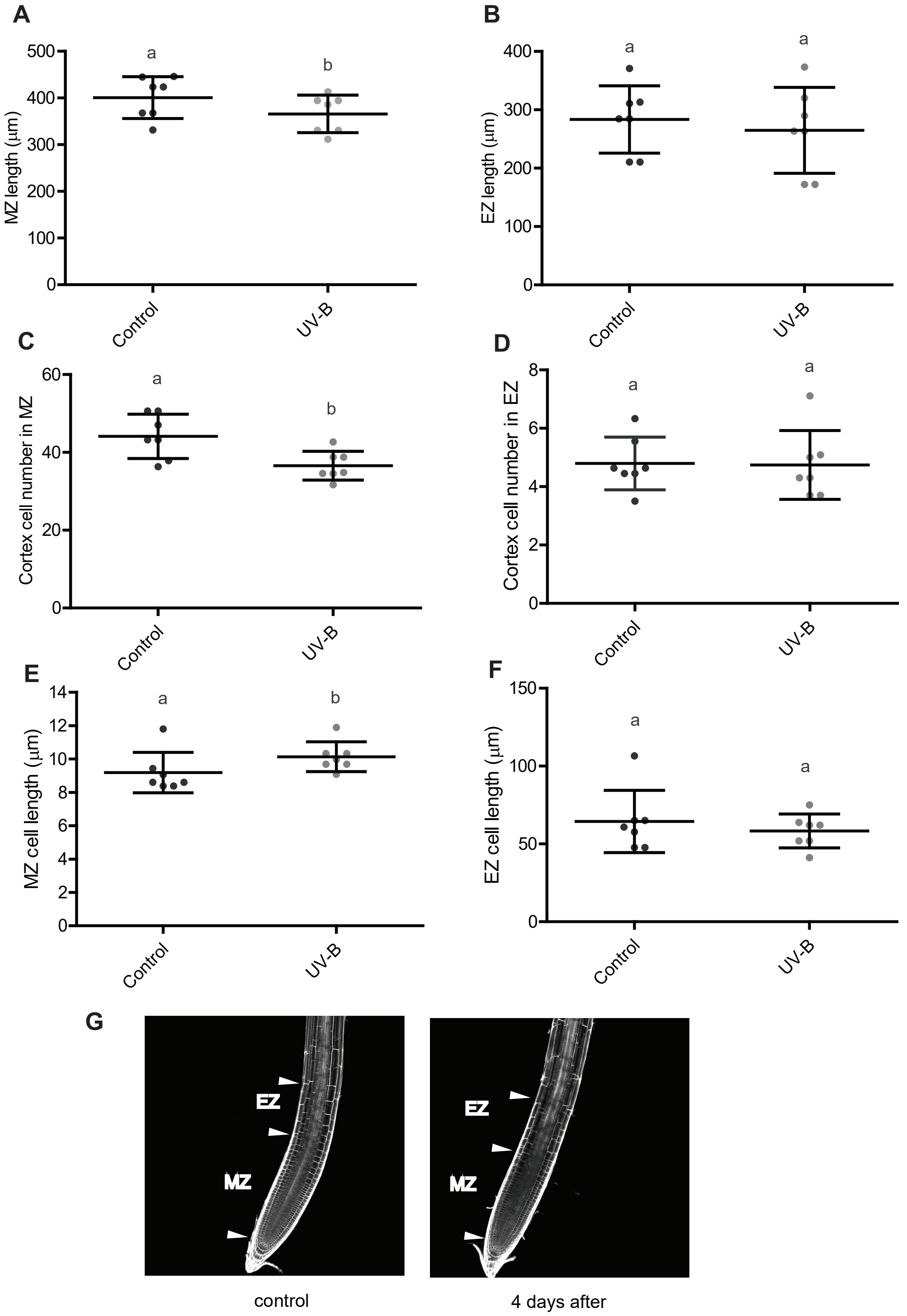

Supplement: Supplementary file 2 [file Image_1.pdf]
