# Supplementary material for: Ultraviolet-B Radiation Represses Primary Root Elongation by Inhibiting Cell Proliferation in the Meristematic Zone of Arabidopsis Seedlings
Source: Front Plant Sci. 2022 Mar 24;13:829336. doi: 10.3389/fpls.2022.829336 (PMC8988989; doi:10.3389/fpls.2022.829336)

SUPPLEMENTARY FIGURE S3 UV-B similarly inhibits cell proliferation in the primary root meristematic zone of WT Col-0 and *mpk3* seedlings.

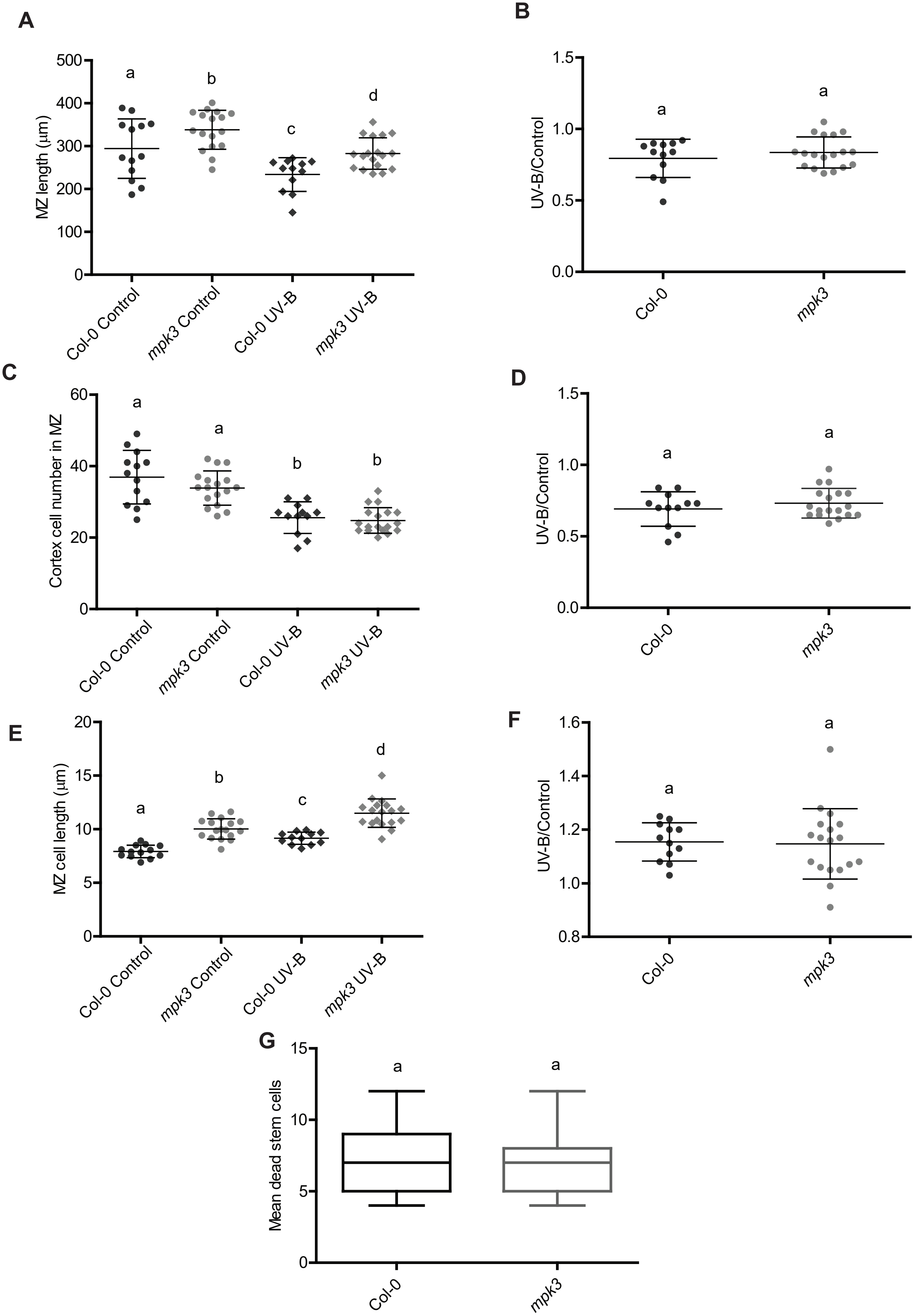

Supplement: Supplementary file 4 [file Image_3.pdf]
