# Supplementary material for: Ultraviolet-B Radiation Represses Primary Root Elongation by Inhibiting Cell Proliferation in the Meristematic Zone of Arabidopsis Seedlings
Source: Front Plant Sci. 2022 Mar 24;13:829336. doi: 10.3389/fpls.2022.829336 (PMC8988989; doi:10.3389/fpls.2022.829336)

SUPPLEMENTARY FIGURE S4 UV-B similarly inhibits primary root elongation in WT Col-0 and *msh6* seedlings recovered under white light or under dark conditions.

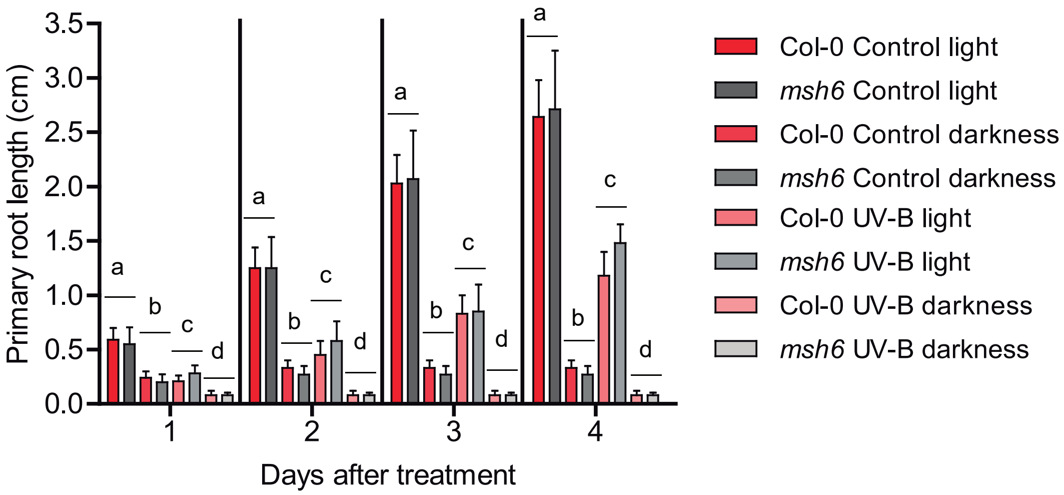

Supplement: Supplementary file 5 [file Image_4.pdf]
